# Characterization of Novel Nonobese Type 2 Diabetes Rat Model with Enlarged Kidneys

**DOI:** 10.1155/2019/8153140

**Published:** 2019-07-31

**Authors:** Ayaka Domon, Kentaro Katayama, Yuki Tochigi, Hiroetsu Suzuki

**Affiliations:** Laboratory of Veterinary Physiology, School of Veterinary Medicine, Faculty of Veterinary Science, Nippon Veterinary and Life Science University, Tokyo 180-8602, Japan

## Abstract

A variety of animal models of diabetes mellitus (DM) are required to study the genetics and pathophysiology of DM. We established a novel rat strain showing nonobese type 2 diabetes with enlarged kidneys from the LEA.PET-*pet* congenic strain and named it Diabetes with Enlarged Kidney (DEK). The body growth of DEK affected rats was similar to that of normal rats before the development of DM but was attenuated with the deterioration of DM. There was a marked difference in the etiology of DEK by gender: DM phenotypes including polyuria, polydipsia, and hyperglycemia (nonfasting blood glucose over 300 mg/dl) were found in male rats aged over 10 weeks but not in female rats. The cumulative incidence of DM in DEK males at the age of 30 weeks was 44.8%. Oral glucose tolerance tests showed glucose intolerance and decreased insulin secretion in response to glucose loading in affected males, features which were exacerbated with age. Affected males exhibited disorganized architecture of pancreatic islets, decreased numbers of *β* cells, and markedly decreased expression of insulin, despite no pathological findings of hemorrhage or infiltration of inflammatory cells in the pancreatic islet. Age-related islet fibrosis appeared similar in normal and affected males. Affected males also showed enlarged kidneys with dilation of renal tubules in both the cortex and medulla, but no obvious glomerular lesions typical of diabetic nephropathy (DN) at the age of 30 weeks. Plasma levels of urea nitrogen and creatinine were normal, but hypoalbuminemia was detected. These pathophysiological features in affected males indicated that their renal function was almost maintained despite severe DM. Taken together, these findings indicate that the affected males of the DEK strain are a novel nonobese type 2 diabetes rat model useful for studying the mechanisms underlying *β* cell loss and identifying genetic factors protective against DN.

## 1. Introduction

Diabetes mellitus (DM) is a metabolic disease characterized by chronic hyperglycemia. The number of DM patients worldwide was approximately 415 million in 2015 and is estimated to reach 642 million by 2040 [1]. In general, chronic hyperglycemia may lead to multiple complications including diabetic nephropathy (DN), which is a leading cause of chronic kidney diseases in humans [2]. Following microvascular and neuropathy complications, DN is the third most common complication with 27.9% of diabetic patients showing DN [3].

DM is largely classified into type 1 diabetes (T1D), type 2 diabetes (T2D), and others (e.g., Maturity-Onset Diabetes of the Young (MODY)) [4]. While T1D is characterized by absolute deficiency of insulin resulting from autoimmune destruction of pancreatic *β* cells, T2D is characterized by insulin resistance and/or relative insulin deficiency [5, 6] and is associated with a variety of pathophysiological events including inflammation, oxidative stress, and ER stress [7–10]. T2D is the most common form of DM and accounts for 90% of all diabetes patients [11]. T2D is a multifactorial disease resulting from interactions between genetic and environmental factors. It was proposed that there is an ethnic/racial difference in genetic predisposition and susceptibility to environmental risk factors for the development of T2D in humans [12]. Since the etiology and phenotype of DM are heterogenous in humans, a variety of animal models of DM are required to study the genetics and pathology of DM and diabetic complications [13].

We unexpectedly found a male rat with polydipsia, polyuria, and hyperglycemia in the LEA.PET-*pet* congenic strain showing high postnatal lethality, thymic hypoplasia, and dwarfism in an autosomal recessive manner [14]. Using this male rat as the founder, we established a novel rat strain showing nonobese type 2 diabetes with enlarged kidneys. We named this diabetic rat strain Diabetes with Enlarged Kidney (DEK). In the present study, we characterized the phenotype of affected rats by comparing them with normal (nondiabetic) rats of the same strain.

## 2. Materials and Methods

### 2.1. Animals

One male rat with polydipsia, polyuria, and hyperglycemia (nonfasting blood glucose level: >600 mg/dl) was found among the offspring, born by cesarean section, of an LEA.PET-*pet* congenic strain rat. This hyperglycemic male rat was initially crossed with an LEA female to obtain F_1_ progeny born by natural delivery. Thereafter, sister-brother mating has been continued between normal females and hyperglycemic males in each generation. Glucose levels were measured in blood from the tail vein using GLUCOCARD MyDIA (Arkray Inc., Kyoto, Japan). Rats exhibiting levels over 300 mg/dl of nonfasting blood glucose were classified as affected rats. All rats were housed in conventional conditions under a 14 : 10 h light : dark cycle with *ad libitum* access to water and standard chow (CR-LPF, Oriental Yeast Co. Ltd., Tokyo, Japan). Room temperature and humidity were maintained at 20 ± 2°C and 50 ± 10%, respectively [14, 15]. Their body weight was measured weekly, with nonfasting blood glucose levels measured between 1 and 7 pm at the ages of 5, 10, 15, 22, and 30 weeks. All animal experiments were approved by Animal Care and Use Committee of Nippon Veterinary and Life Science University and were performed in accordance with the Guidelines of the Animal Care and Use Committee of Nippon Veterinary and Life Science University.

### 2.2. Oral Glucose Tolerance Test (OGTT)

Oral glucose tolerance test (OGTT) was performed as described [16] with some minor modifications. Briefly, after 16 h fasting, rats were given 2 g/kg BW of glucose orally and their glucose levels were measured in blood from the tail vein at 0, 30, 60, 120, 180, and 240 min after glucose loading. Area under the curve (AUC) of blood glucose levels were calculated. Blood collected from the jugular vein under isoflurane anesthesia at 30, 60, 120, and 240 min after glucose loading was mixed with heparin and centrifuged for 15 min at 4°C to obtain plasma to measure insulin concentration. Plasma insulin concentrations were measured using an LBIS Rat Insulin ELISA Kit (RTU) (Shibayagi, Gunma, Japan).

### 2.3. Insulin Tolerance Test (ITT)

Insulin tolerance test (ITT) was performed as described [17] with some minor modifications. After fasting for 4 h, rats were intraperitoneally administered 0.5 U/kg Humulin R (Eli Lilly Japan K.K., Hyogo, Japan) then blood glucose levels were measured at 0, 30, 60, 120, and 180 min.

### 2.4. Histological and Immunohistological Analysis

Tissue samples collected from euthanatized rats aged 15 and 30 weeks were weighed with an analytical balance then fixed in neutral buffered 10% formalin solution or 4% paraformaldehyde in phosphate-buffered saline (PBS). The samples were rinsed with PBS, dehydrated, and embedded in paraffin following standard procedures [14, 15]. Samples were then sectioned 4 *μ*m thick for hematoxylin and eosin (H&E), periodic acid-Schiff (PAS), and Masson's trichrome staining and immunofluorescence. Kidneys were also sectioned into 1.5 *μ*m for periodic acid methenamine silver (PAM) staining. For antigen retrieval, sections were incubated with 10 mM citrate buffer at 121°C for 5 min. Sections were blocked with 3% bovine serum albumin (BSA) in PBS and incubated with primary antibodies overnight at 4°C. The primary antibodies used were guinea pig anti-insulin polyclonal (1 : 100, Abcam K.K., Tokyo, Japan) and mouse anti-glucagon polyclonal (1 : 200, Abcam K.K., Tokyo, Japan). After rinsing with PBS, sections were incubated with Alexa Fluor 488 goat anti-guinea pig IgG antibody (1 : 1000, Life Technologies, Carlsbad, CA) or Alexa Fluor 568 donkey anti-mouse IgG antibody (1 : 1000, Life Technologies, Carlsbad, CA) for one hour at room temperature and mounted with the ProLong Gold Antifade Reagent with DAPI (Life Technologies, Carlsbad, CA). Images were acquired with an all-in-one fluorescence microscope (Biozero BZ-X800) (KEYENCE, Tokyo, Japan).

### 2.5. Calculation of Insulin-Positive Area

Pancreas sections were rehydrated, blocked with 3% BSA in PBS for 30 min, then incubated with 0.3% H_2_O_2_ in PBS to inactivate any endogenous peroxidase activity. Sections were next incubated with guinea pig anti-insulin polyclonal antibody (1 : 100) overnight at 4°C, rinsed with PBS, and incubated with horseradish peroxidase- (HRP-) conjugated goat anti-guinea pig antibody (1 : 400, Santa Cruz Biotechnology Inc., Santa Cruz, CA) for one hour at room temperature. Immunoreactivity was visualized with 3,3′-diaminobenzidine (DAB) substrate (BD Pharmingen, San Diego, CA), and insulin-positive areas were measured using ImageJ software.

### 2.6. Semiquantitative RT-PCR and Sequence Analysis

Total RNAs were extracted from pancreas with the Trizol Reagent (Life Technologies, Carlsbad, CA), and cDNAs were synthesized using ReverTra Ace and oligo d(T)20 primer (TOYOBO, Osaka, Japan). Densitometric analysis of RT-PCR for *Ins1* and *Ins2* was performed with ImageJ software and normalized by the *β*-actin amplification level of the respective sample. Entire coding sequences of *Ins1* and *Ins2* genes were sequenced with amplified cDNA fragments. Genomic DNAs were extracted from blood using the ZymoBead Genomic DNA Kit (Zymo Research, Irvine, CA). All exon and exon/intron boundaries of the *Hnf1β* gene were amplified from genomic DNA and sequenced.

### 2.7. Measurement of Metabolic Parameters

Following habitation for 2 days, rats were housed in metabolic cages for 24 h, and urine volume, drinking water volume, and amount of food intake were measured [18]. The concentrations of creatinine (Cre) and glucose (Glu) in urine were measured with Dri-Chem 3500V (FUJIFILM, Tokyo, Japan). Protein concentration in urine was measured using the Protein Assay Rapid Kit *Wako* II (FUJIFILM Wako Pure Chemical Corporation, Tokyo, Japan). Total amounts of Cre and protein excreted within 24 h were determined. After 24 h housing, blood was collected from the jugular vein under isoflurane anesthesia, and plasma concentrations of urea nitrogen (UN), Cre, albumin (Alb), total protein (TP), total cholesterol (Tcho), and Na-K-Cl were measured with Dri-Chem 3500V [19].

### 2.8. Measurement of Blood Pressure

Blood pressure was measured using a BP-98A blood pressure system (Softron, Tokyo, Japan). Blood pressure was taken in triplicate per rat, and individual averages were calculated [18].

### 2.9. Statistical Analysis

The values were presented as means ± standard deviations (SD). To compare between normal and affected rats, two-tailed Student's *t*-test was performed with *P* < 0.05 considered statistically significant.

## 3. Results

### 3.1. Gender Difference and Incidence of DM

**Figure 1:**
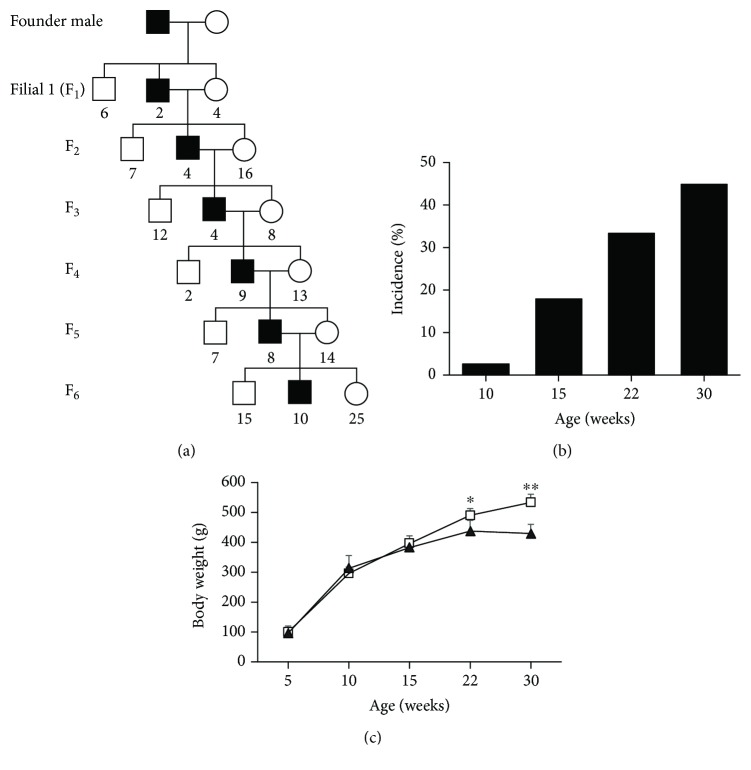
(a) Pedigree of DEK (Diabetes with Enlarged Kidney) strain rats. Squares and circles indicate males and females, respectively. Open and closed squares indicate nondiabetic (normal) and affected diabetic males, respectively. Numbers of descendants with normal or affected phenotypes are indicated at the bottom of each shape. (b) Cumulative incidence of affected rats in males of the strain (*n* = 78). (c) BW of normal and affected males. Squares and triangles represent normal and affected males, respectively. ^∗^*P* < 0.05 and ^∗∗^*P* < 0.01.

We found one male rat with severe hyperglycemia (nonfasting blood glucose level > 600 mg/dl) among the offspring of an LEA.PET-*pet* congenic strain rat born by cesarean section. In the present study, we defined rats showing levels of nonfasting blood glucose over 300 mg/dl as affected. When the founder affected male described above was crossed with a female from the LEA strain, two affected males and four females phenotypically normal without DM resulted. Subsequent brother-sister mating between affected males and normal females produced affected males in each generation but no affected female, indicating a gender difference in this inherited DM (Figure 1(a)). Nonfasting blood glucose levels measured in rats 5, 10, 15, 22, and 30 weeks old revealed that DM developed from the age of 10 weeks, and the cumulative incidence of DM in males was 44.8% at the age of 30 weeks (Figure 1(b)).

### 3.2. Growth and Body Weight

**Figure 2:**
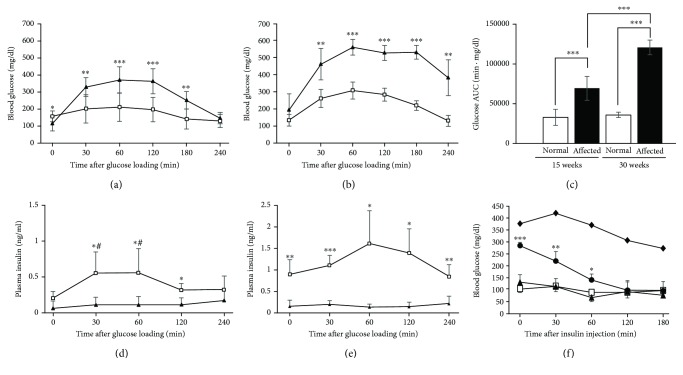
Blood glucose levels after glucose loading in normal (square) and affected (triangle) males aged 15 ((a), normal *n* = 16, affected *n* = 7) and 30 ((b), normal *n* = 5, affected *n* = 5) weeks. (c) Areas under the curve (AUC) of blood glucose after glucose loading at the ages of 15 and 30 weeks. Open and closed bars represent normal and affected males, respectively. Plasma insulin levels after glucose loading in normal (square) and affected (triangle) males aged 15 ((d), normal *n* = 7, affected *n* = 4) and 30 ((e), normal *n* = 4, affected *n* = 4) weeks (e). (f) Insulin tolerance test at the age of 22 weeks. Affected rats were classified into three groups (triangle, *n* = 5; circle, *n* = 3; rhombus, *n* = 2). Normal rats are depicted by open squares. ^∗^*P* < 0.05, ^∗∗^*P* < 0.01, and ^∗∗∗^*P* < 0.001: normal vs. affected. ^#^*P* < 0.05: vs. 0 min.

Body weight (BW) was measured weekly, and BW data was classified into the normal group or the affected group, retrospectively. Normal and affected males showed similar growth until the age of 15 weeks from when an increase in BW of affected males was attenuated and was significantly lower than that of normal males at ages of 22 and 30 weeks (Figure 1(c)). This lower increase in BW of affected males was concomitant with the deterioration of DM (Figure 2). Necropsy of affected males showed the absence of adeps renis at the age of 30 weeks (data not shown). The data of growth and necropsy demonstrated that affected males developed nonobese DM.

### 3.3. Oral Glucose Tolerance Test (OGTT) and Intraperitoneal Insulin Tolerance Test (ipITT)

Rats were orally loaded with glucose (2 g/kg BW) after 16 h fasting to characterize glucose tolerance and insulin secretion. Affected males showed glucose intolerance and significantly high levels of blood glucose from 30 to 180 min after glucose loading compared to normal males at the age of 15 weeks (Figure 2(a)). At the age of 30 weeks, affected males showed more severe glucose intolerance, with blood glucose levels significantly higher from 30 to 240 min after loading, than normal males (Figure 2(b)). Values of area under the curve (AUC) of blood glucose levels in affected males significantly increased with age (Figure 2(c)), indicating exacerbation of DM with age.

Plasma insulin levels significantly increased in response to glucose loading in normal males at the age of 15 weeks (Figure 2(d)) compared with affected males, all of which exhibited significantly low levels of plasma insulin (Figure 2(d)). This unresponsiveness of plasma insulin to glucose loading was also found in affected males at the age of 30 weeks (Figure 2(e)).

To examine the response to insulin, we performed an intraperitoneal insulin tolerance test (ipITT) in rats aged 22 weeks after 4 h fasting. Based on blood glucose levels before intraperitoneal insulin injection and response to exogenous insulin, affected males were classified into three groups (Figure 2(f)). One group (*n* = 5, triangle in Figure 2(f)) showed blood glucose levels and response to exogenous insulin similar to normal males. Another group (*n* = 3, circle in Figure 2(f)) exhibited significantly higher blood glucose levels than normal males, which decreased to levels similar to those in normal males at 120 min after insulin injection. A third group (*n* = 2, rhombus in Figure 2(f)) showed markedly high blood glucose levels (338 and 413 mg/dl) after 4 h fasting. Rats in the third group showed almost no response to exogenous insulin, and blood glucose levels did not decrease to those of normal male rats even 180 min after insulin injection (Figure 2(f)).

### 3.4. Pathology of Pancreas and mRNA Expression of Insulin Gene

**Figure 3:**
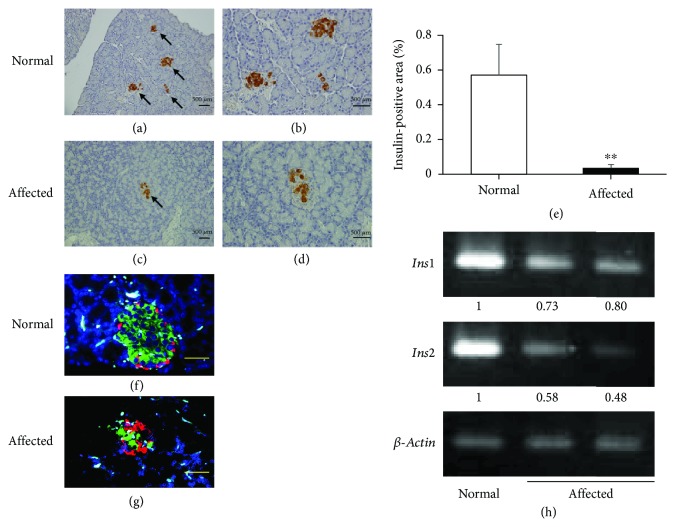
Immunohistochemistry of insulin in normal pancreas (a and b) and affected (c and d) pancreas in 15-week-old rats. (e) Percentage of insulin-positive area in pancreatic sections of normal (open bar, *n* = 3) and affected (closed bar, *n* = 3) male rats aged 30 weeks. Immunofluorescence of insulin (green) and glucagon (red) of normal pancreas (f) and affected (g) pancreas in rats aged 15 weeks. (h) Semiquantitative RT-PCR of *Ins1* and *Ins2* genes in normal pancreas and affected pancreas in rats aged 30 weeks. Relative intensities of amplified fragments are represented. Scale bar = 50 *μ*m. ^∗∗^*P* < 0.01.

To characterize any pathological changes in the pancreas of affected males, we performed histological and immunohistological analyses at the ages of 15 and 30 weeks. We found no hemorrhage or obvious inflammatory cell infiltration in the pancreatic islets at either age. In addition, we observed no difference in pancreatic fibrosis between normal and affected males aged 30 weeks evaluated by Masson's trichrome staining (data not shown). Immunostaining for insulin indicated reduced numbers of pancreatic islets with insulin immunoreactivity in the affected males (Figures 3(a) and 3(c)). The insulin-positive area in an affected pancreas was significantly smaller than that in a normal pancreas (Figure 3(e)). A lower intensity of insulin immunostaining was observed in affected islets as compared to those observed in normal islets (Figures 3(b) and 3(d)). In normal pancreatic islets, *α* cells (glucagon-positive cells) were located at the periphery, whereas *β* cells (insulin-positive cell) were located in the center of the islets (Figure 3(f)). This localization of *α* and *β* cells was severely disorganized in the pancreatic islets of affected males aged 15 and 30 weeks (Figure 3(g) and data not shown). Accompanying these pathological changes, expression levels of *Ins1* and *Ins2* genes were markedly reduced in affected males (Figure 3(h)).

### 3.5. Metabolic Parameters and Blood Pressure

Normal and affected males were individually housed in metabolic cages at the age of 30 weeks. Food intake, water intake, and urinary volume during 24 h were measured and all were found to be significantly higher in affected than in normal males (Table 1). To evaluate renal function and metabolic status in affected males, biochemical parameters in urine and plasma were measured. Total amounts of glucose (Glu) excreted into urine during 24 h were significantly higher in affected than in normal males. Protein excretion was higher in the urine of affected males compared to that of normal males, albeit not significantly, and there was no observable difference in Cre excretion (Table 2). Among the biochemical parameters in plasma, concentrations of albumin, total cholesterol (Tcho), and chloride were significantly lower in affected than in normal males (Table 3). Other biochemical parameters in plasma including creatinine and urea nitrogen (UN) did not differ (Table 3), nor did heart rate and systemic blood pressure, between normal and affected males (Table 4).

### 3.6. Pathology of Kidney

**Figure 4:**
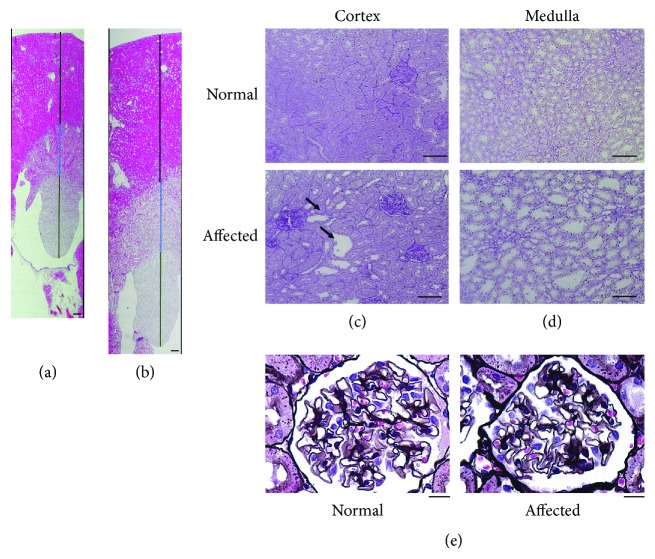
Renal histology at the age of 30 weeks. Joint images of sections stained with HE in normal (a) and affected (b) kidneys. Black, blue, and green depict the length of the cortex, medulla, and renal papilla, respectively. PAS staining of cortical (c) and medullary (d) sections in normal and affected kidneys. Arrows indicate dilated renal tubules in the cortex of affected kidneys. (e) PAM staining of juxtamedullary glomeruli in normal and affected kidneys. Scale bars: 300 *μ*m (a and b), 100 *μ*m (c, d), and 50 *μ*m (e).

We found that affected males had markedly larger kidneys than normal males at the age of 30 weeks, with absolute and relative weights being 1.5- and 1.9-fold higher in affected than in normal males, respectively (Table 5). The kidneys of affected males showed marked enlargement at all regions, including the cortex, medulla, and renal papilla (Figures 4(a) and 4(b)). Some dilated tubules were observed in the cortex of affected kidneys (arrows in Figure 4(c)). In contrast, most renal tubules in the medulla showed marked dilation in affected kidneys (Figure 4(d)). We found no glomerular lesions characteristic of DN such as glomerular hypertrophy, glomerulosclerosis, or mesangial expansion (Figure 4(e)).

## 4. Discussion

In the present study, we found a male rat with DM symptoms among rats in the LEA.PET-*pet* congenic strain. We crossed that rat with a normal female of the LEA strain and bred a novel diabetic strain. We characterized the phenotype of affected offspring rats and named the strain DEK. The *pet* locus is responsible for dwarfism, thymus hypoplasia, and postnatal semilethality [14, 15]. We mapped the locus on rat chromosome 9 using microsatellite markers and confirmed that the DEK phenotype is independent of the *pet* genotype (data not shown). The LEA rat strain (LEA/SENDAI) has been characterized as a mild type of nonobese diabetes model [16]. Although our DEK strain has an LEA genetic background, the phenotypic features of DEK differ substantially from those of LEA/SENDAI. DEK rats showed more severe glucose intolerance compared to LEA/SENDAI; e.g., our affected rats aged 30 weeks exhibited plasma glucose levels >400 mg/dl at 120 min after oral glucose loading, whereas it was reported that 1-year-old LEA/SENDAI only exhibited >200 mg/dl [16]. In addition, our histological analyses of the pancreatic islets in affected males detected no pathological features such as inflammatory cell infiltration which has been reported in LEA/SENDAI [16]. These characteristic differences between DEK affected and LEA/SENDAI rats may be attributed to a substrain difference. LEA/SENDAI has been maintained in Tohoku University Graduate School of Medicine [16], while DEK includes a genetic background of LEA strain rats maintained in the Institution of Animal Reproduction (Ibaraki, Japan).

A remarkable gender difference was found in the DEK phenotype, as only male rats developed DM. Some reports have suggested that estrogen and testosterone have protective effects against DM [20, 21]. However, ovariectomy in female littermates of DEK affected males did not induce any symptoms of hyperglycemia (*n* = 11, data not shown). Furthermore, there was no significant difference in the weight of male accessory genitalia, which are target organs of testosterone, between normal and affected males. Therefore, the gender difference in the incidence of DEK is not simply explained by sex steroid hormones. Although the gene responsible for the DM of DEK are unknown at this time, pedigree clearly shows that DEK have genetic predisposition for DM, and our preliminary genetic analysis using an outcross with Wistar rats suggests that the DM of DEK is assumed to be polygenic trait (data not shown).

In general, DM is categorized into three groups: type 1, type 2, and others (e.g., Maturity-Onset Diabetes of the Young (MODY)) [4]. To date, a variety of spontaneous diabetes animal models have been established. NOD mouse and BBDP rat are both type 1 DM animal models characterized by autoimmunity with the infiltration of mononuclear cells into the pancreatic islet [22, 23]. Akita mouse is a type 1 DM animal model that shows *β* cell dysfunction and progressive loss of *β* cells caused by ER stress due to a point mutation of the insulin 2 (*Ins2*) gene [24, 25]. Type 2 DM is categorized into two types: obese and nonobese type 2 DM. Animal models of obese type 2 DM, including ZDF rat [26], *db* mouse [27], and KK mouse [28], typically exhibit hyperglycemia, hyperinsulinemia, and insulin resistance. In contrast, animal models of nonobese type 2 DM such as Goto-Kakizaki (GK) rat [29] and Spontaneously Diabetic Torii (SDT) rat [30] typically exhibit hyperglycemia and hypoinsulinemia rather than insulin resistance. GK rats exhibit moderate hyperglycemia, islet inflammation, and fibrosis [31]. SDT rats show severe hyperglycemia, gender difference (cumulative incidence: 100% in male and 33% in female), and hemorrhage in pancreatic islets leading to the infiltration of inflammatory cells into the islets and islet fibrosis [32]. In the present study, two affected males showed insufficient response to exogenous insulin (Figure 2(f)). However, the principal cause of DM in the affected rats of the DEK strain seems to be hypoinsulinemia, because all affected males examined showed markedly low plasma insulin levels and a decrease in pancreatic *β* cells. We found no hemorrhage or inflammatory cell infiltration in the pancreatic islets of affected males. In addition, we observed a similar degree of fibrosis in the pancreatic islets of both normal and affected rats aged 30 weeks. Therefore, pancreatic fibrosis in affected males seems to be an age-related histological feature [33] rather than the cause of DM. We also confirmed that affected rats had no mutation in the coding regions of *Ins1* and *Ins2* genes (data not shown). Since it has been reported that decrease of the pancreatic *β* cell is associated with a variety of pathophysiological events including ER stress and oxidative stress [9], further experiments are required to elucidate the mechanism underlying the decrease of pancreatic *β* cells in affected males. Nevertheless, our findings clearly show that the phenotype of affected males in the DEK strain is different from those of other DM model animals previously reported. Therefore, we conclude that affected rats in the DEK strain are a novel nonobese type 2 DM animal model with decreased pancreatic *β* cells, hypoinsulinemia, severe hyperglycemia, a remarkable gender difference, and enlarged kidneys.

All the affected males examined in this experiment showed significantly enlarged kidneys. In addition, we found one male and one female rat exhibiting a polycystic kidney at the F_3_ and F_6_ generations, respectively. Therefore, we sequenced the entire coding sequence and exon/intron boundaries of the Hnf-1*β* gene, which is responsible for MODY5 exhibiting diabetes and renal malformation in humans, and found no mutation in the Hnf-1*β* gene of affected rats in the DEK strain (data not shown). DN is one of the complications in diabetes and a leading cause of chronic kidney diseases in humans [34]. To date, various DN animal models have been studied to elucidate the mechanisms and genetic factors involved in the development and progression of DN [13, 35]. DN initially causes kidney enlargement commonly associated with glomerular hypertrophy, and after that, renal function declines with the reduction of renal size in humans and rodents [36–39]. Severity of DN in rodents varies with strain and genetic background [38]. GK rats have mild phenotypes for DN among type 2 diabetic rodent models [38], but they exhibit increased plasma UN and creatinine levels at the age of 10 weeks [40] and increased urinary albumin excretion at the age of 2 months [41]. The DEK affected males aged 30 weeks showed decreased levels of plasma total cholesterol. In contrast, other nonobese T2D rat models, such as GK and SDT rats, showed a high plasma total cholesterol level compared to normal rats [32, 42]. This difference implies that DEK might have a unique lipid metabolism even though the underlying mechanism remains unclear. The DEK affected males aged 30 weeks also showed decreased levels of plasma albumin and chloride, probably due to their leakage into urea (Table 3). Hypoalbuminemia is an important predictor of stages of renal dysfunction becoming more severe at the end stage of renal failure [43], and hypochloremia occurs in metabolic alkalosis during polyuria. However, we found no obvious glomerular lesions in affected kidneys. In addition, plasma levels of UN and creatinine, biochemical parameters for renal function, did not differ between normal and affected rats (Table 3). And plasma levels of potassium and sodium other than chloride were almost the same in normal and affected rats, suggesting that there was no collapse in electrolytic balance. Taken together, the pathophysiological features associated with DN are very mild, and renal function is almost fully maintained in affected rats of the DEK strain. In a preliminary study, increased renal parenchyma were observed in affected rats. Therefore, it is likely that the increased renal parenchyma contributes to renal enlargement and retention of renal function. This suggests that DEK affected rats have protective genetic factors against the progression of DN as well as genetic predisposition for DM.

## 5. Conclusion

We established a novel nonobese diabetic rat strain (DEK) characterized with polyuria, polydipsia, hyperglycemia, disorganized pancreatic islet architecture, loss of pancreatic *β* cells, and enlarged kidney with nearly normal renal function. The DEK rats will be useful for studying the mechanism of pancreatic *β* cell loss and for identifying protective genetic factors against DN.

## Figures and Tables

**Table 1:** Food intake, water intake, and urinary volume during 24 h of normal (*n* = 8) and affected (*n* = 10) males aged 30 weeks. ^∗∗^*P* < 0.01.

	Food intake (g)^∗∗^	Water intake (g)^∗∗^	Urinary volume (ml)^∗∗^
Normal (*n* = 8)	26.9 ± 5.5	31.1 ± 8.7	14.8 ± 5.6
Affected (*n* = 10)	47.0 ± 6.0	168.9 ± 42.5	143.6 ± 34.8

**Table 2:** Protein, creatinine (Cre), and glucose (Glu) excreted during 24 h into the urine of normal (*n* = 3) and affected (*n* = 3) males aged 30 weeks. Data are presented as mean ± SD with ^∗∗∗^*P* < 0.001.

Parameters	Normal (*n* = 3)	Affected (*n* = 3)
Protein (mg/day)	12.9 ± 8.6	34.1 ± 33.6
Cre (mg/day)	21.6 ± 9.1	18.0 ± 1.3
Glu (mg/dl)^∗∗∗^	35.3 ± 11.2	8840.0 ± 695.6

**Table 3:** Biochemical parameters in plasma of normal (*n* = 4) and affected (*n* = 4) males aged 30 weeks. Data are presented as mean ± SD with ^∗^*P* < 0.05 and ^∗∗^*P* < 0.01.

Parameters	Normal (*n* = 4)	Affected (*n* = 4)
Tcho (mg/dl)^∗^	108.8 ± 5.6	81.3 ± 16.4
Cre (mg/dl)	0.43 ± 0.05	0.43 ± 0.05
UN (mg/dl)	15.0 ± 2.7	18.1 ± 6.7
TP (g/dl)	5.7 ± 0.39	5.0 ± 0.48
Alb (g/dl)^∗∗^	4.1 ± 0.17	3.6 ± 0.19
Na (mEq/dl)	139.5 ± 3.8	134 ± 4.2
K (mEq/dl)	4.9 ± 0.35	5.9 ± 1.1
Cl (mEq/dl)^∗^	100.5 ± 4.4	93.3 ± 3.3

**Table 4:** Systemic blood pressure of normal (*n* = 4) and affected (*n* = 7) males aged 30 weeks. Data are presented as mean ± SD. HR: heart rate; SBP: systolic blood pressure; MBP: mean blood pressure; DBP: diastolic blood pressure.

Parameters	Normal (*n* = 4)	Affected (*n* = 7)
HR	406.5 ± 22.6	374.1 ± 39.1
SBP	117.1 ± 16.2	134.0 ± 21.9
MBP	100.3 ± 12.8	110.4 ± 16.8
DBP	92.1 ± 11.1	98.8 ± 16.6

**Table 5:** Kidney weights of normal (*n* = 10) and affected (*n* = 16) males aged 30 weeks. Relative kidney weights are shown as kidney weight (mg) relative to BW (g). Data are presented as mean ± SD with ^∗^*P* < 0.05 and ^∗∗^*P* < 0.01.

	Normal (*n* = 10)	Affected (*n* = 16)
Absolute weight (mg)^∗∗^	2079.8 ± 401.6	3234.9 ± 841.2
Relative weight^∗^	3.9 ± 1.0	7.6 ± 1.9

## Data Availability

The authors confirm that the data supporting the findings of this study are available within the article.
